# Effect of post-discharge postnatal educational intervention on postnatal practices among low-income primiparas in Nairobi informal settlements, Kenya: a post-test quasi-experiment

**DOI:** 10.11604/pamj.2024.48.14.42194

**Published:** 2024-05-16

**Authors:** Immaculate Wambui Kamau, Margaret Nyanchoka Keraka, Eliphas Gitonga

**Affiliations:** 1 Department of Environmental and Occupational Health, School of Health Sciences, Kenyatta University, Nairobi, Kenya

**Keywords:** Low-income, primiparous mothers, primiparas, postnatal care, postnatal education, informal settlements

## Abstract

**Introduction:**

informal settlements exhibit disproportionately worse maternal and newborn outcomes. Postnatal care (PNC) is a high-impact intervention for populations contributing to higher mortalities. Postnatal education is crucial to adopting evidence-based postnatal practices (PNPs) thus its post-discharge reinforcement is vital for low-income primiparas. This study aimed to determine the effect of post-discharge follow-up postnatal education intervention (PNE) on the adoption of recommended PNPs among low-income primiparas.

**Methods:**

quasi-experimental study in Nairobi informal settlements with 118 primiparas discharged early after normal delivery on each arm. Facility and demographic data collected using an interviewer-administered questionnaire. Study arm received the intervention for 6 weeks in addition to routine PNC while control received routine PNC only. An interviewer-administered exit questionnaire was applied after 6 weeks. Focus group discussions were conducted for qualitative data then analyzed thematically. SPSS used to analyze quantitative data then descriptive statistics, t-tests, Chi-square, Mann-Whitney, and multiple linear or logistic regressions derived. PNPs composed of health-seeking for maternal and newborn danger signs, self and baby care practices, and utilization of PNC contacts.

**Results:**

the intervention was a positive predictor of adoption of composite PNPs (β=0.26, p=0.00), self-care practices (β=0.39, p=0.00) and mothers' two weeks PNC contact (OR=4.64, p=0.00, 95% CI=1.9-11.2). Neither a significant predictor of health-seeking for maternal (β=-0.11, p=0.31) nor newborns danger signs (β=-0.04, p=0.73) though inversely related. No influence on baby care practices, two weeks of newborn contact and six weeks contact for the dyad. Multi-pronged approaches are appreciated.

**Conclusion:**

follow-up post-discharge PNE intervention enhances adoption of PNPs among low-income primiparas thus a worthwhile supplement to routine PNC.

## Introduction

Maternal and neonatal mortalities remain global health concerns. The sustainable development goals (SDGs) 3.1 and 3.2 aim to reduce the maternal mortality ratio (MMR) to less than 70 per 100,000 live births, and the neonatal mortality rate (NMR) to 12 per 1,000 live births by 2030 [1]. Kenya is more than fivefold behind with an MMR of 362 per 100,000 live births [2] and almost 2-fold in NMR at 22 deaths per 1,000 live births [2]. The informal settlements in Nairobi contribute disproportionately higher MMR at 709 per 100,000 live births [3]. Similarly, NMR in Nairobi- where 60-70% of the population resides in the slums [3] is at 39 per 1000 live births [2]. Most of these deaths are from preventable causes [4].

The postnatal period (PP) is risky for both the mother and the baby as it is characterized by unacceptably high mortalities [5]. Unfortunately, strategies designed to reduce MMR and NMR and endorsed by the World Health Organisation (WHO) such as PNC remain underutilized [5,6]. Christened the poor Cinderella of midwifery for decades [7], PNC is inadequately considered compared to antenatal and intrapartum, especially in sub-Saharan Africa (SSA [8]. Pre-discharge information provision, educational interventions, and counseling to new mothers are some of the PNC WHO recommendations to enhance maternal and newborn health outcomes and ease the transition [5]. Postnatal education (PNE) is key to the adoption of evidence-based practices and maternal confidence in transitioning home [9,10]. It improves maternal knowledge (MK) thus promoting healthy postnatal practices (PNPs) hence reducing adverse outcomes. For instance, educating women to recognize postnatal danger signs and when to seek urgent medical help saves lives [11]. Similarly, timely PNE improves the use of evidence-based newborn care practices and timely healthcare seeking for illnesses thus saving newborn lives [10]. Indeed, pre-discharge PNE with post-discharge reinforcement has proven helpful in promoting maternal and newborn health in Low and Middle-Income Countries (LMICs) [10]. Unfortunately, delivery of in-hospital PNE is adversely affected by the short hospital stay for normal delivery, limiting the time available compounded by a large amount of information and a shortage of staff [9,12-14]. Consequently, the literature highlights unmet maternal information needs among women [15] and thus inadequate MK in low-resource settings [16,17]. In Nairobi County Kenya, only 49% of mothers had adequate MK on discharge [18].

Unfortunately, the provision of hospital-based post-discharge PNE is curtailed by challenges such as limited knowledge of the essence of PNC services, insufficient staffing, inadequate infrastructure, and sub-optimal scheduling of PNC contacts [19,20]. This leaves the mothers exposed to the risks of low MK throughout the PP. Low MK is associated with low; household income, education, and social support and leads to low Maternal Self Efficacy (MSE) and poor early parenting practices [16] which leads to poor parental outcomes [21]. These challenges are more accentuated among primiparas whose inexperience predisposes them to a stressful transition into motherhood compounded by a lack of continuity of care following early discharge [9,21]. Low-income status further exacerbates these challenges. Indeed, young mothers who are socioeconomically disadvantaged have complex informational needs made worse by the effects of poverty [22]. Sadly, there has been limited focus on low-income women despite their diverse needs and lack of follow-up after early discharge [23]. This calls for innovative educational interventions beyond the hospital door especially for the primiparas.

Evidence favors multimodality programs that combine experience with the practice of skills, question, and answer, M-health technology, and individualized sessions with content presented via diverse formats such as videos, activities, and up-to-date printed self-reading resources [24,25]. Also, the key is incorporating early postpartum home visits by health professionals or community health volunteers (CHVs) in low-resource settings [5,26]. Interventions that cover multiple practices, especially in LMIC are recommended [10] for their cost-effectiveness.

Though follow-up PNE interventions are proven impactful for primiparas and neonatal outcomes in low-resource settings, little is known about their effect on the adoption of evidence-based PNPs among low-income primiparas living in informal settlements in Kenya. This study determined the effect of post-discharge follow-up educational intervention on the adoption of key WHO-recommended PNPs including health-seeking for maternal and neonatal danger signs, self-care and baby-care practices, and attendance of PNC healthcare contacts for the mother and the baby at two and six weeks postpartum among low-income primiparas in informal settlements in Nairobi County, Kenya. We hypothesized that the intervention group would have significantly enhanced adoption of the recommended PNPs at 6 weeks compared to the control group.

## Methods

**Study design and setting:** a quasi-experimental study, post-test-only design applying quantitative and qualitative data methods was conducted in Nairobi informal settlements. Korogocho and Viwandani slums were purposefully selected having been the two demographic surveillance areas providing urban poor health data in the Nairobi Urban Health and Demographic Surveillance System (NUHDSS) since 2002 [3] thus providing us with the requisite indicators. The small target population meeting our inclusion criteria based on 2019 deliveries in the targeted facilities necessitated the expansion of the study area by adding adjacent slums; Huruma to Korogocho (experimental site) and Kwa Reuben to Viwandani (control site). The study period was between June and December 2021.

**Participants:** health facilities offering maternity services to the selected slums as per the Nairobi County guidance were purposefully included in the study. Given the rarity of the target population, convenience sampling was applied. The discharge list for the day was obtained from the nurse in charge from which consenting primiparas who had uncomplicated birth to a healthy singleton, discharged within 48 hours, who hoped to be domiciled in the study location for 2 months postpartum, had access to a telephone and could be visited by a CHV at home were recruited on a rolling basis until the desired sample was reached. The experimental participants were discharged as per the routine facility criteria and connected to a CHV for the 6 weeks - postnatal period- intervention in addition to routine PNC while the control group received routine MoH PNC only which comprises facility-based contacts at 2 weeks but mostly at 6 weeks with no home-based follow-up. Participants of focus group discussion (FGDs) were purposefully selected after the 6-week follow-up based on consent and availability.

### Variables

**Independent variables**

**Facility factors:** type of facility ownership (private, public, FBO), level of facility (level 2-6), postnatal ward workload ratio, length of hospital stay (LOHS) after delivery, and perceived satisfaction with pre-discharge PNE.

**Social demographic factors:** age, occupation, level of education, household income (later dropped due to low response), and marital status.

**Intervening variable (experimental):** follow-up PNE intervention.

**Dependent variable:** adoption of PNPs a composite score of 8 self-reported components: emergency health-seeking for maternal danger signs (MDSs) and NDSs; self-care and baby care practices, two and six weeks contact for the neonate and the mother.

**Figure 1:**
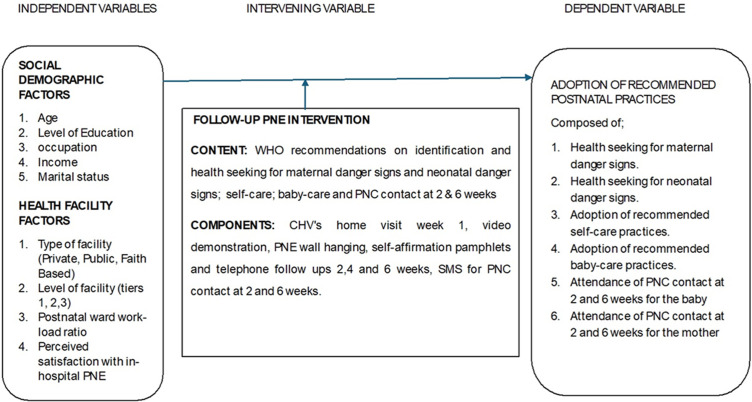
the conceptual framework

**The intervention:** comprised WHO-recommended PNE content including the identification and health-seeking for maternal danger signs (MDSs) and neonatal danger signs (NDSs), baby care practices, self-care practices and the utilization of PNC contacts at two and six weeks. Content delivery included: i) the specially trained CHVs to deliver the 6-week follow-up christened “adopt a primi”; ii) a home visit by the CHV in the first week to provide 45-minute didactic teaching with demonstrations of basic baby care tasks such as bathing the baby, cord care, and breastfeeding; iii) a video of a similar mother (from *Mathareslum*) demonstrating key baby care skills while being directed by a nurse was used and later uploaded on the mother/significant others´ phone for reference. Extra videos on newborn care, self-care, MDSs, and NDSs from the Global Health Media Project [27,28], were used for reinforcement; iv) presence of significant others during the home visit sessions for support and affirmation; v) a PNE wall hanging in a convenient place for constant reinforcement; vi) motherhood self-affirmation pamphlets in both English and Kiswahili; vii) the CHVs called the mothers three times at two, four, and six weeks for any concerns; viii) the CHVs sent short message (SMS) reminders for PNC visits a day before 2 and 6 weeks; ix) 24-hour telephone access to the CHVs for any troubleshooting (Figure 1).

**Data sources:** interviewer-administered questionaries were used to collect entry data; facility data from maternity in-charges, perceived satisfaction with in-hospital PNE, and social demographic data from the participants while an exit questionnaire was applied to collect PNPs data after the 6-week intervention. Focus group discussion guides were used to collect qualitative data and notes were taken concurrently.

**Bias:** to avoid researchers' bias different RA´s were used for the groups. To control the recall and desirability bias the PI sampled and re-interviewed random respondents with minor differences.

**Study size:** the sample size was calculated according to Chan (2003) formula for the comparison of two proportions two-sided [29] at a 5% significance level and 80% statistical power. Since we could not determine the mean PNP adoption rates in the study population, the generally agreed 50% [30] was assumed, with a 20% effect size yielding 91 participants per group. Based on a 30% dropout rate in a previous study [31], 118 participants were required for each arm. Qualitative data was collected from 2 FGDs per arm with eight participants each. Groups were coded as Viwandani E1 and Kwa Reuben E2; Korogocho C1 and Huruma C2 with participants allocated no.1-8 to enable coding of participants by group and number.

**Figure 2:**
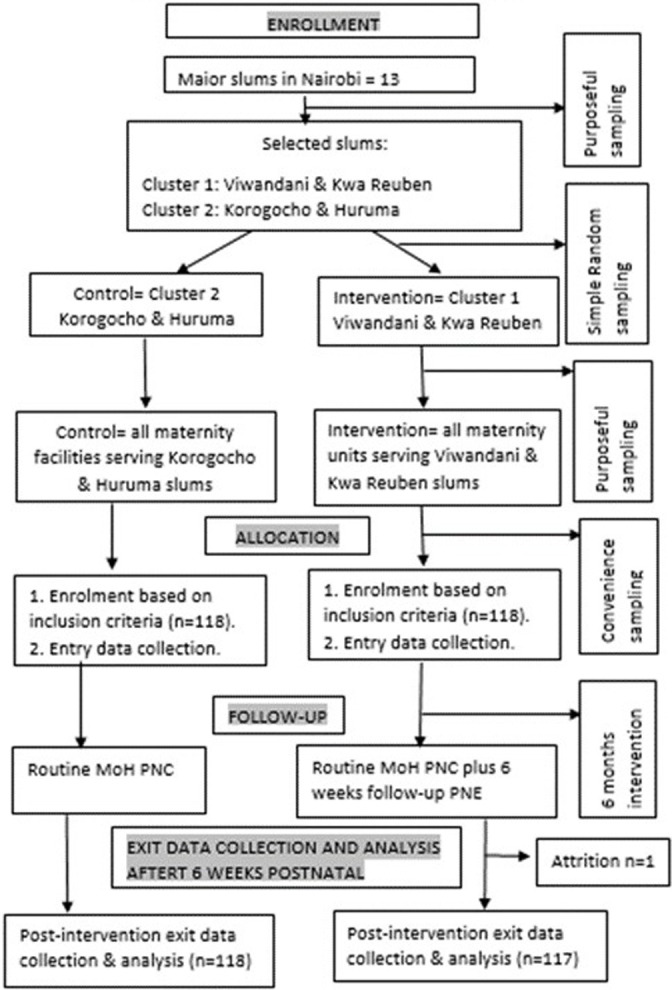
study flow chart of the intervention

**Quantitative data analysis and statistical methods:** data were entered into the Open Data Kit (ODK) and then exported to IBM SPSS Version 20.0 for analysis. Descriptive statistics such as percentages, means and standard deviations were derived for facility, demographic characteristics, and PNP scores. To test the difference between the groups t-test for continuous data or Mann-Whitney test for non-parametric data was calculated. To isolate the effect of the intervention, hierarchical multiple linear for continuous data or logistical regressions for nonparametric data were conducted and adjusted for covariates. The difference was considered significant at P<0.05. There was one loss to follow-up in the experimental group after losing the newborn thus their entire data were eliminated reducing the respondents to 117 (Figure 2). The response rate for household income was extremely low at 28% (65), likely due to COVID-19-related loss of livelihoods, thus omitted from the analysis. Though questionnaires were checked for completeness in real time, any cases with missing data were omitted from the analysis.

**Qualitative data analysis:** the content analysis method was used where common responses were identified, color-coded, labeled, and meanings deduced in line with postnatal practices, views on support, associated challenges, and desired improvements.

**Logistical and ethical considerations:** Kenyatta University Ethical Review Committee granted the ethical clearance approval PKU /2173/11317. A research permit no. NACOSTI/P/21/9084 was granted by the National Commission for Science, Technology, and Innovation (NACOSTI). Written permission was obtained from the Director of health services, Nairobi County. Informed written consent was obtained from each respondent. Confidentiality was maintained by not using identifiers but rather codes. Written permission was obtained from the Global Media Project to use their videos. Electronic data was password protected while scripts were locked away by the principal investigator. Scripts are to be shredded and electronic storage overwritten on completion of the PhD study.

## Results

The respondent's background characteristics are reported elsewhere [32]. There was a statistically significant difference between the groups on facility type; facility level; marital status; occupation, postnatal ward workload ratio, and LOHS. The adoption of recommended PNPs was an additive composite variable and thus the risk of losing individual variables´ information necessitated the analysis of the eight self-reported components. The statistical difference between the groups was derived followed by the effect of the intervention adjusted for all the covariates (education level, occupation, age, marital status, facility level, facility type, postnatal workload ratio, satisfaction with pre-discharge teaching, and LOHS to avoid variable omission bias (Table 1).

**Table 1:** comparison of danger sign incidences and subsequent health seeking between the control and experimental groups

Statistical differences between the groups					Effect of the intervention adjusted for covariates				
**Outcome variable**	**Group**	**N**	**Mean (SD)**	**Statistical test**	**P-value**	**R^2^**	**β/OR**	**P-value**	**CI (95%)**
**Total maternal danger signs incidences reported per mother**									
	Experimental	(n=117)	0.74(1.11)	t=-9.5	p=0.00*	0.42	-0.59	0.00*	
	Control	(n=118)	2.26(1.35)						
**Respondents that reported to have had incidences per group**									
	Experimental	(n=117)	0.44(0.5)	t=-7.3	p=0.00*	0.31	0.036 (OR)	0.00*	[0.009-0.151]
	Control	(n=118)	0.85(0.36)						
**Proportion that sought health care after MDS incidences**									
	Experimental	(n=51)	0.86(0.33)	t=-2.8	p=0.01*	0.165	-0.11	0.31	
	Control	(n=100)	0.97(0.13)						
**Total neonatal danger signs incidences reported per newborn**									
	Experimental	(n=117)	0.74(1.05)	t=-10.56	p=0.00*	0.4	-0.54	0.00*	
	Control	(n=118)	2.49(1.46)						
**Total newborns reported to have had incidences per group**									
	Experimental	(n=117)	0.48(0.50)	t=-6.67	p=0.00*	0.31	0.193(OR)	0.00*	[0.076-0.49]
	Control	(n=118)	0.86(0.35)						
**The proportion that sought health care after NDS incidences**									
	Experimental	(n=56)	0.95(0.23)	t=-0.62	p=0.53	0.194	-0.036	0.73	
	Control group	(n=102)	0.97(0.16)						

N: number, std. dev: standard deviation, R^2^: R square, β: standardized coefficient, *: p-value ≤ 0.05, CI: confidence interval

**Emergency health-seeking for maternal danger signs (MDSs):** to determine MDS health-seeking adoption rates, incidences of 8 key MDSs were determined as a process indicator. The intervention was protective where the odds of experiencing MDSs was 0.036 in the experimental compared to the control group. The difference in health-seeking was statistically significant (t=-2.8, p=0.01), with less uptake in the experimental group. However, after adjusting for covariates, the intervention was not a significant predictor (β=-0.11, p=0.31) though the inverse relationship was sustained. Some of the reasons were that accessibility of the CHVs reduced health-seeking for the mothers from the intervention arm while some were pacified by their significant others. Lack of knowledge also contributed to the failure to seek care in the control group. “*My breasts were painful and cracked but I called the CHV who told me to massage with a hot towel and apply vaseline*”, E1.7 “*I was just feeling weak, lacked strength and tired but was told by my friends its kawa (normal)*” E2.1. “*Sikuona kama kukua na low moods na kulia ni kitu serious. I didn´t go*” (I did not know that having low moods and crying is serious), C2.5.

**Emergency health-seeking for neonatal danger signs:** incidences of 10 key NDSs were determined as a process indicator. The intervention was protective with 0.193 odds of experiencing NDSs in the experimental group. There was a statistically significant difference in reported NDSs per baby (t=-10.56, p=0.00). Almost all (96% experimental, 94% control) sought immediate healthcare after NDS with no statistically significant difference (t=-0.62, p=0.53). After adjusting for covariates, the effect on health-seeking for NDSs was not statistically significant, (β=-0.04, p=0.73).

**Figure 3:**
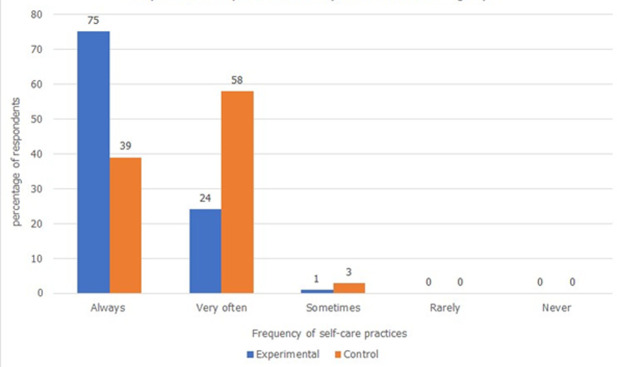
comparison of adoption of self-care practices by respondents in experimental and control groups (experimental n=117, control n=118)

**Self-care practices:** thirteen self-care practices were evaluated using a four-point Likert scale. The experimental group had better scores (Figure 3). There was a statistically significant difference in the adoption of recommended self-care practices (U=4380, p=0.00) between the groups. To enable a regression analysis, data were numerically transformed by calculating the means of each respondent. The intervention predicted 39% adoption adjusted for covariates. Hindrances to some self-care practices were mostly tied to slum life difficulties such as the inability to rest due to lack of help with house chores and inability to obtain good nutrition. “*I can't rest, though I want I can't always be able to buy good food*”, C2.4. “*Kukosa kibarua kulifanya nikose chakula nika-lose weight sana na nikakosa maziwa*”, C2.8. (I didn´t get casual jobs so I lacked food and breast milk),

**Baby care practices:** seven baby care practices were evaluated. There was no statistically significant difference (t=0.44, P=0.23) between the groups. After adjusting for covariates in a multiple regression analysis, the PNE intervention was not a significant predictor (β=.021, p=0.80). Challenges related to insufficient income were highlighted as affecting the adoption of some baby care practices such as exclusive breastfeeding. “*Because of lack of enough food, I didn't have breast milk for the baby so I gave her shop milk*”, E1.4.

**Utilization of postnatal care (PNC) contact at two and six weeks:** there was a statistically significant association between the adoption of two weeks of newborn PNC contact (χ^2^=7.6, df=1, p=0.006) and the study arm which was lost after adjusting for covariates in a logistic regression (p=0.98). There was a statistically significant association between the adoption of two weeks' mother´s PNC contact (χ2=20.9, df=1, p=0.00) and the study arm. At six weeks, there was no significant association for both the baby and the mother. The intervention was a significant predictor of two weeks postpartum contact (p=0.00) where the mothers in the experimental group were 4.6 times more likely to attend, but not a significant predictor of the six weeks contact (p=0.69) after adjusting for covariates (Table 2).

**Table 2:** comparison of utilization of newborn and mothers' PNC contact at 2 and 6 weeks between the groups

			Difference between groups			Effect of intervention adjusted for covariates			
**Variable**	**Group**	**n**	**Chi-square**	**df**.	**P-value**	**R^2^**	**OR**	**P-value**	**CI (95%)**
**2 weeks visit for the newborn**									
	Experimental	117	7.6	1	0.006*	0.62	0.95	0.98	[0.017-51.98]
	Control	118							
**6 weeks visit for the newborn**									
	Experimental	117	2	1	0.158	1	0	0.996	[0.00-0.00]
	Control	118							
									
**2 weeks visit for the mothers**									
	Experimental	117	20.9	1	0.00*	0.18	4.64	0.00*	[1.93-11.19]
	Control	118							
**6 weeks visit for the mothers**									
	Experimental	117	0.001	1	0.973	0.13	1.22	0.69	[0.47-3.13]
	Control	118							

N: number, std. dev: standard deviation, R^2^: R square, β: standardized coefficient, *: p-value ≤ 0.05, CI: confidence interval

**Adoption of composite postnatal practices:** the principle of proportionality was applied to calculate the fraction score for each of the eight components which was averaged into a composite score per respondent. There was a statistically significant difference (t=8.1, p=0.00) between the groups. After adjusting for covariates, 26% of the variance was predicted by the intervention. This demonstrated that compared to the control group, mothers in the intervention group had statistically significant enhancement of adoption of the recommended PNPs, thus accepting the alternative hypothesis. More specifically, the intervention had a positive effect on self-care practices and 2 weeks postnatal contact for the mother (Table 3).

**Table 3:** summary of the adjusted effect of PNE intervention on composite and individual PNP variables (n=235)

Outcome variables	Test statistic (multiple linear/logistic regression)	P-value
Composite postnatal practices	β= 0.26	0.002*
MDS health-seeking	β =-0.107	0.31
NDS health-seeking	β =-.036	0.73
Self-care practices	β= 0.385	0.00*
Baby-care practices	β =-0.002	0.98
2 weeks visit for the newborn	OR= 0.945	0.98
6 weeks visit for the newborn	OR =0.0	0.996
2 weeks PNC contact (mother)	OR= 4.64	0.00*
6 weeks PNC contact (mother)	OR= 1.22	0.69

* : P≤0.05, OR odds ratio, β: beta coefficient

Upon the intervention, most respondents reported better satisfaction and appreciated the diverse content delivery methods, especially CHVs. “*I was happy with my CHV coming home. She taught me a lot, I asked many questions, and she showed me what to do*”, E2.6. “*For me calling a CHV, videos and the encouraging pamphlets were the best any time in doubt*”, E1.1. On the contrary, most mothers from the control group expressed the need for professional follow-up. “*Tunahitaji kufunzwa na nurse nyumbani kwasababu hatuwezi kumbuka vile tulifunzwa hosi*”. ( we need post-discharge teaching by a nurse because we cannot remember the teaching received in the health facility), C1.7. “*We need references at home on sicknesses to know what is serious and what is not*” C2.6.

Though an unforeseen phenomenon, some COVID-19-associated challenges affected the mothers. They highlighted the loss of livelihood affecting the affordability of basic commodities and restrictions on social connectedness denying them the much-needed social support. “*Life is hard with COVID, getting a job is hard and I have no enough food*”, C2.4. “*Lockdown affected visitors. Mum couldn´t travel, furaha ya mama nikutembelewa”, (a mother´s joy is being visited*), C2.3. The CHV visit was especially appreciated in light of COVID-19 isolation.

## Discussion

Our general finding is that follow-up PNE intervention enhanced the adoption of recommended PNPs. Besides mitigating information loss, the analysis of the adoption of individual PNP components also enabled comparison with similar studies given that most focus on one outcome [10]. In this study, health-seeking after experiencing MDS was better in the control group than in the experimental. Though not significant, the intervention had an unexpected inverse effect on health-seeking. This could be because of the access to the CHVs with studies showing that such follow-up reduces maternal anxiety [33] and improves maternal outcomes without increasing utilization of health services [34]. The intervention may have improved the accurate identification of danger signs and/or the CHV´s pacified the mother's concerns, thus reducing unnecessary health-seeking. Home visits by CHVs have been shown to reduce unnecessary hospital visits for other conditions [35]. Although this may reduce healthcare costs, caution is necessary to ensure health-seeking when essential.

Over 94% of all respondents sought emergency health care for NDSs. This was better than in Ethiopia where only 60.5% of the mothers sought health care immediately after an NDS. Contrary to our study where the intervention was not a significant predictor, Bulto *et al*. [36] reported that those who had PNC follow-up had better health-seeking. Both groups in our study may have received routine emphasis on seeking care for the newborns thus the high and similar uptake despite the intervention. The inverse relationship, though not significant may have implied that the intervention quelled unwarranted health-seeking.

Both groups scored impressively in the adoption of baby-care practices though the intervention was not a significant predictor. This is congruent with a study in Afghanistan which showed no improvement in newborn care practices after CHV home visits [37]. This was however contrary to a study in India where topic-specific PNE follow-up enhanced newborn care practices [10]. The self-reported, prompted recall of practices in this study may have been subject to desirability bias thus no significant difference. Similar unprompted studies have demonstrated effectiveness thus still a worthy intervention. In fact, home visits by CHVs promote neonatal care practices and are cost-effective in enhancing newborn health outcomes in LMICs [38]. Our intervention being a significant positive predictor of self-care practices was congruent with a similar multi-modal follow-up study in Iran which demonstrated that an early self-care-based education program is effective for primiparas´ outcomes [39]. It was also congruent with a study in Nigeria where women who received teaching on self-care of perineal wounds had better practices [40]. Targeting maternal self-care was a strength in our study since a recent study identified a gap not only in maternal self-care education but also in its suboptimal attention from researchers [14].

The intervention being neither a significant predictor of the two nor six weeks of contact for the newborn may be due to routine emphasis on the baby´s check-up making the indicator less amenable to the intervention. This is congruent with a study in Oman that showed that health workers scheduled and emphasized on two-week review for the baby and not the mother, then six weeks for both [19]. Unsurprisingly, our intervention positively predicted the mother´s contact at two weeks, but not at six weeks. Though most respondents said they had not been scheduled for the two-week PNC contact, the experimental group had a significantly higher uptake, presumably due to the SMS and call reminders. Indeed, research affirms that SMS and telephone call reminders increase the uptake of PNC visits in developing countries [41]. Comparable uptake of six weeks of contact in both groups could be attributed to routine emphasis on the baby´s immunization and the mother´s family planning, thus less amenable to intervention. Indeed, healthcare workers emphasize the six weeks of contact more than two weeks for the mothers [19]. Failure to schedule mothers for PNC contact before six weeks portrays the two-week visit as non-essential and optional affecting uptake [20]. The workload is cited as the main deterrent [20] which underscores the need to sufficiently resource PNC to mitigate its non-prioritization and to supplement it with home-based interventions [19].

According to WHO, CHVs are key players in improving maternal health indicators in low-resource settings [5]. Tasking the CHVs to “adopt a primi”, teach, support, and follow the primiparas through the PP was a great strength. It was the most appreciated aspect by primiparas living in the harsh informal settlements. This agrees with the evidence that young mothers appreciate home visits by professionals as they assist them with recognizing and addressing their needs [22]. A significant other was included in the CHV visit where they were encouraged to support and affirm the primiparas. Indeed, research shows that postpartum mothers appreciate and benefit from home visits especially if the sessions involve the influential caregivers and decision-makers in the family [42]. For most respondents, the CHVs were the only social support they had occasioned by COVID-19 restrictions where mothers expressed feeling isolated. Irrefutably, home-based PNE support, telephone follow-up, self-affirmations, educative posters, and verbal feedback worked together to enhance the adoption of PNPs. The findings demonstrate that the use of multi-modal approaches in follow-up PNE is helpful for low-income primiparas. This is in tandem with other studies that favor post-discharge reinforcement using multifaceted teaching approaches [10,24,25].

Having multiple practices in this study was a strength in keeping with the recommendation that it is more cost-effective to cover a variety of evidence-based practices in PNE programs in LMICs [10]. We believe that the findings of our study contribute to the WHO´s call for a positive postnatal experience by providing women, newborns, and families information, reassurance, and support in their homes [5]. The limitations occasioned by the onset of COVID-19 and the rarity of our target population increased the cost and duration of the study. The observed outcomes were self-reported and thus may have been subject to desirability bias. Analysis of each of the components of the composite PNPs increased the volume of data.

Overall, the study findings are a useful insight for PNC service providers in developing innovative follow-up strategies for low-income primiparas. The intervention can be adopted into routine care. The findings contribute to the body of knowledge for researchers who can further interrogate the interventions´ effect on primiparas with longer LOHS and those who have complicated births. A cost-benefit analysis of scale-up would be helpful. Replications of the study in different informal settlements preferably using a randomized controlled trial (RCT) may help ascertain generalizability.

## Conclusion

The follow-up post-discharge PNE intervention enhanced the adoption of PNPs among low-income primiparas, specifically, the adoption of self-care practices and the two-week PNC contact for the mother. Incidentally, the intervention showed the potential to reduce unnecessary healthcare visits. Primiparas appreciated multi-pronged sources of information, especially the early PP educational home visits by the CHVs. The intervention can be a worthwhile supplement to routine PNC given that improving the adoption of evidence-based PNPs upstream improves maternal and newborn outcomes downstream thus contributing towards SDGs 3.1 and 3.2.

### What is known about this topic

- *Primiparas suffer knowledge and skill gaps that negatively impact their adoption of evidence-based postnatal practices;*
- *Home-based postnatal follow-up has the potential to improve primiparas outcomes*.

### What this study adds

- *Multimodality home-based follow-up PNE enhances the adoption of WHO-recommended postnatal practices among primiparas living in informal settlements*;
- *During emergencies such as COVID-19, home-based care can be helpful postnatal support*;
- *Follow-up care by CHVs has the potential to lower unnecessary health-seeking among low-income primiparas*.
